# Bodily integrity and autonomy of the youngest children and consent to their healthcare

**DOI:** 10.1177/14777509231188006

**Published:** 2023-07-27

**Authors:** Priscilla Alderson

**Affiliations:** Social Research Institute, University College London, London, UK

**Keywords:** Best interests, competence, diabetes care, heart surgery, refusal

## Abstract

Children's autonomy includes, as far as possible, self-determination, bodily integrity and the right to influence outcomes. Limits to bodily integrity, which involves no touching without the child's consent or tacit agreement, are discussed. The clinical, legal and ethics literature tends to agree that children may give valid consent to major recommended treatment from around 12 years but may not refuse it until they are legal adults. Research shows that young children are more aware of their bodily integrity and autonomy, of morality and decision making, than was assumed in the past. Adults therefore need to inform children and respect their initially instinctive efforts to protect their bodily integrity. Unlike assent, consent involves patients being adequately informed and being able to accept or refuse proposed treatment. Reasons are given for adults’ need to consult with children when determining their best interests. Beyond words, giving or withholding consent also involves emotions of fear, trust and courage, besides embodied reactions of cooperating with treatment or resisting it, in which young children actively engage. Some clinicians work with the informed cooperation of young children who need lifesaving treatment, and at times accept their refusal. Reasons for differences between mainstream experts’ views and clinical practices are considered.

## Three sentences on how the paper is helpful

The paper considers research on young children's physical, mental, emotional and moral awareness, which relates to their bodily integrity and autonomy.

The need for adults to inform children and respect their consent or refusal is discussed.

Reasons are given on why children should be involved in determining their best interests, and why mainstream experts’ views in law and ethics lag behind clinical practice.

## Introduction

The literature about children's bodily integrity and autonomy in relation to consent to their healthcare is based on certain views. These include: how far young children are aware of, understand, and are concerned about their bodily integrity and autonomy; children's best interests and parents’ rights and legal responsibilities; children's rational, informed decision-making capacity; and consent as primarily a legal or a personal process. This paper questions dominant older views in the mainstream clinical, legal and ethics literature and contrasts these with newer thinking and with research examples of children's understanding and decisions. Reasons for differences between the dominant older published views and children's actual reported experiences will be considered.

Most of the children we studied were unusual in being deeply informed by their severe problems when living with chronic illness or disability, and by their previous treatments. This paper is less relevant to children facing new problems without related experiences, and to emergencies, and controversial contested types of treatment. After a summary of traditional literature, newer insights and findings about children's bodily integrity and autonomy, their awareness, best interests and decision making are presented.

## Children's awareness of their bodily integrity and autonomy

In law, bodily integrity, the body being undivided and not touched without consent, is the first and ‘most important of the civil rights’.^1^ Physical interference with bodily integrity requires greater justification than is needed for (mental) interference with autonomy.^2^ For example, a doctor who refuses to provide treatment that a patient has requested only interferes with the patient's autonomous choice, but a doctor who performs an intervention the patient has refused also interferes with that person's bodily integrity, which requires stronger justification in law. Bodily integrity (of adults of sound mind) is an unquestionable right, whereas autonomy is a provisional right limited by unlawful exercise of autonomy. Bodily interference is only lawful when the patient's competence is in question, such as with minors or in cases of severe mental illness or incapacity, and when the person's best interests are respected.

Mervi Patosalmi contrasts two versions of bodily integrity.^3^ Nussbaum's version separates mind and body, so that bodily integrity means physical autonomy and inviolability.^4^ In Cornell's version, mind and body are inseparable; bodily integrity involves protecting how the whole present and future self and personal autonomy are interpreted, imagined and presented.^5^ This relates to Feinberg's moral concern to protect children's opportunities for their fulfilled ‘open future’.^6^ Bodily autonomy involves self-governing awareness and ability to control the activities and safety of the body. Integrity and autonomy are mainly associated with intellectually informed, adult-centric dignity and independence.^7^

For centuries, the law treated children as if they were their father's mindless property subject to his control. English law, for example, did not recognise children's need to be protected from abusive parents until 1889.^8^ Psychologists assumed the baby cannot think or process experience but is overwhelmed by sensations and ‘feels it all as one great blooming, buzzing confusion’.^9^ Anna Freud thought children were ‘unrestrained, cruel and greedy little savages’ not yet ‘civilized human beings’.^10^ ‘Theory of mind’ research reports that children begin to ascribe mental states to other persons and distinguish their own self from others at around 4, 3, or possibly 2 years.^11^ It was long assumed that young children had no sense of having a personal identity, integrity or autonomy. In the 1980s, anaesthetists believed that new-born babies have no memory and therefore have no awareness of their bodies or of pain.^12^ New-born babies who had surgery were administered curare (to paralyse them) and anaesthesia (to induce unconsciousness) but not analgesia (pain relief).

Key words here are ‘mind’ and ‘cognition’. Following Descartes's mind/body split, researchers have assumed that young children cannot understand concepts such as self-awareness or bodily integrity or autonomy, until (1) they are verbally informed about these concepts by adults and (2) the child can think through and verbally explain these concepts.^13^

However, every day and research evidence challenge these views. On pain, a clinical trial in 1989 showed that new-born babies who were given analgesia recovered better from surgery. They need pain relief.^14^ New-born babies turn towards the sound of their parents’ voices, preferring them to other voices and remembering them from before birth.^15^ Babies are calmed and soothed when gently spoken to. They become agitated, cry and resist if suddenly handled by silent strangers and their anxiety is ignored. Premature babies in hospital quickly learn to flinch when their foot is held, anticipating the heel prick blood test, and they are very distressed by the pain from the invasive needle.^16^ From their first months, babies are meaning makers, connecting cause to effect, and intuiting adults’ intentions.^17^ Like other young animals, human babies’ instinctive self-preservation involves trying to avoid danger and pain, and to enjoy nurturing care.

Babies appear to be highly aware of practical differences between their own ‘mind’ and the ‘minds’ of those who care for them in their conflicting desires. For example, they protest against being put into a cot if they want to be held or fed. They cry persistently in a battle of wills, which they often win. Babies intuit others’ intentions when they fix onto the proffered bottle or breast and start feeding, and they soon learn to hold out their arms ready to be picked up or dressed. Instinctive reactions quickly develop into subtle responses. Fifteen-week-old babies take part and laugh in response to simple games, and also initiate repetitive games.^18^ Babies’ first words include naming other people whom, from their early weeks, they have recognised, relating differently to each individual adult and child they know.

When ‘evidence’ about babies’ capacities extends beyond words to include observed behaviours, babies’ rich awareness of their bodily integrity and autonomy can be seen through their interactions, choices and preferences, clear reactions to pleasure or pain, and obstinate determination. Instead of recognising the child's autonomy only when it is clearly developed in cognitive and verbal terms, adults can observe: young children's gradually developing embodied autonomy; their efforts to protect their bodily integrity; and the benefits of cooperating with young children whenever possible to avoid forcing and coercing them.

## Children's best interests and parents’ rights and legal responsibilities

In the tradition that saw children starting as “blank slates”^19^ gradually filled by adult instruction, children cannot know their best interests. It was assumed that ignorant, foolish, volatile children must be controlled by informed, wise, responsible adults. Adult-centric experts claim that parents have the right to decide freely for their children.^20^ Competence to consent involves the wisdom to know one's best interests, and the courage to accept responsibility if a choice is mistaken or the outcomes are harmful.^21^ Children have long been assumed to be too immature to be wise or courageously responsible. Their response to proposed surgery is frequently termed ‘assent’. However, assent has been termed ‘conceptually hollow’;^22^ it does not require that patients are adequately informed or have the option to refuse proposed treatment.

One view of children's best interests promotes protecting their ‘open future’, preventing any choices or actions that might limit future options.^23^ This may imply childhood is like waiting in a long corridor lined with doorways. Children should not enter any rooms that might close their later options to enter other rooms. Clearly, there are doorways to avoid: lifestyles that reject education or that cause self-harm. But engaging quite deeply in any single positive activity involves taking time and opportunity away from others. Many high achieving adults passionately specialised in their work at a very early age, thereby closing doors to other opportunities. ‘Best interests’ therefore involves helping children to choose their options wisely.

Yet parental rights extend only to protecting their child's interests and fulfilling parents’ legal responsibilities.^24^ It is necessary for children's health and wellbeing to listen to children's own views, especially when these differ from adults’ views.^25^

Aoife Daly contends that respect for children's autonomy, which includes their self-determination, bodily integrity, and right to influence outcomes, should be promoted as a priority among children's best interests.^26^ To replace the present weak ‘right to be heard’,^27^ children need rights, embedded in good systems, to be encouraged and assisted to express themselves. All children's views should be respected unless there is significant risk of harm. Autonomy is so highly valued (by and for adults) in liberal democracy because we are ‘rational beings, deserving of dignity and control over our own destinies’ and wellbeing. It is therefore ‘extraordinary that there has been so little progress in negotiating…the adult/child legal dichotomy [which] denies children any legal control over their own persons’, and respect for autonomy therefore involves mutual respect for everyone's dignity and protection and support when needed.^26^

English Gillick law^28^ is ambiguous and outdated; it can adversely affect decisions about minor's treatment in law and clinical practice, so that legal guidance needs to be changed.^29^ Children should not have to pass a test of ‘competence’ or ‘maturity’ before they can be heard, when these terms are used so casually, and the disempowering effects of contexts and relationships on children are usually ignored.^30^ For example, children may remain ignorant because of poor information from adults rather than because of their own inabilities. ‘Competence’ and ‘maturity’ cannot be clearly defined or assessed, in the collusion of ‘pseudo-science’ between lawyers and psychologists, whereas the ‘presumption of capacity, plus protection’ for children can safeguard and nurture their capacity with adequate information and support.^31^

Vague legal understandings of ‘bodily integrity’ should be replaced by an ‘embodied integrity’ model that respects children's rights; this at least ‘should trump competing values in any best-interests assessment where a non-therapeutic intervention is requested…Protecting a child's embodied integrity is essential to guarantee his/her right to make future embodied choices and become a fully individuated person’.^32^ Respect for bodily integrity involves no touching without the child's consent, or tacit agreement with everyday routines, unless the child or others are in danger.

Bodily integrity, when each person has the right to exclusive use and control over his or her own objective body in a combined physical-mental-emotional-objective-subjective security, matters because power over ‘the use of our bodies promotes our wellbeing, flourishing, relationships and agency, these morally valuable states’ that keep the body inviolate.^32^ Our ‘subjectivity (agency, well-being, dignity, and capabilities) are experienced and lived through the body’. Yet human bodily integrity and autonomy are subject to numerous conscious and subconscious influences. We are all interdependent, and experience and express ourselves through sensations and emotions as well as through thoughts. Embodied interactions between persons cause the greatest fear and danger, and also the greatest personal joy and fulfilment. The legal right to autonomous choice before each interaction is therefore vital. These insights can enfranchise children, when respect for intellectual decisions (informed consent that confuses many adult patients) has less weight in law than respect for patients resisting enforced interference with the body (practical consent or resistance that even babies actively perform).

Through knowing their children's hopes and fears, adults can help them to accept choices that they may at first reject. If informed choices result in harmful outcomes, it can be easier for any patients to accept difficult outcomes when they shared in decision making and were prepared about the risks.

## Children's rational, informed decision making and capacity to consent

In the 1970s, a professor of Philosophy of Education claimed that children have no moral awareness until they ‘overcome their passions and self-love’ and begin to reason and respect others when aged from about 7 years.^33^ Recently, however, researchers studied micro-second analysis of videos of babies’ faces while they reacted to puppet shows. The videos revealed that from about 3 months babies are thinking morally about justice and kindness, long before anyone can explain anything to them in words.^34^

Piaget concluded that children cannot understand conservation (continuity over change) until they are about 6 years.^13^ However, researchers who improved on Piaget's research methods found 3-year-olds could understand conservation.^35^ The belief that competence to understand and weigh risks begins at around 12 years echoes Piaget's theory that adolescents start to make probability judgements when they can explain their thoughts verbally.^13^ Yet psychologists now accept that young children know and intuit much more than they can say. Their probability intuitions are highly structured, they can distinguish determining events from chance, and connect outcomes to causes.^36^

The English 1989 *Children Act* takes account of children's ‘wishes and feelings’ and developing ability to make decisions. The 1989 United Nations *Convention on the Rights of the Child,*^24^ adopted and ratified by every country in the world except the USA, also supports children’ involvement, though not their actual decisions.

Lawyers still mainly agree that children have capacity to consent to major recommended treatment from around 12 years (*Gillick* competence in English law, mature minors law in the USA) but they cannot give legally valid refusal until they are adults.^37,38^ Designed mainly by philosophers and lawyers, concepts of consent to healthcare treatment and research centre on words. Patients are informed about the nature and purpose of proposed treatment, the risks, benefits and alternatives, they may ask questions and, if satisfied, they sign the form.^39^ Yet this formal verbal process is only part of consent. Doctors have time to convey little of the complex relevant knowledge, and many adults misunderstand or forget much of it.^40^ Even adult patients’ knowledge is therefore partial, leaving them to trust that doctors are honest and well-intentioned.^41^ Legal standards of consent do not always protect patients or health services. In 2021–2022, the UK NHS paid over £13billion for negligence, including harms to patients after they were not properly informed.

However, formal consent, an essential legal safeguard, helps to protect many patients and doctors from harrowing complaints and litigation. Here, the legal status of the adequately competent, rational, consenting patient or parent is central. Yet other aspects of consent are also vital for all age groups. Consent is an emotional process, moving from fear and possible initial rejection of dangerous procedures, to doubt when weighing risks, hoped-for benefits and alternative (such as no treatment), towards growing trust and confidence in the clinical team, to the courage to be committed and give voluntary (unforced) consent or refusal.^42^ Besides verbal interactions, these emotions and the quality of doctor–patient relations are crucial, even for very young children.

Further, children enact consent with bodily autonomy and integrity when they actively cooperate with treatment or resist it. Many nurses are reluctant to enforce procedures.^43^ To avoid daily battles, even very young children with type I diabetes need to understand that their painful, daily finger-prick blood tests and insulin injections really are in their best interests, and that the adults want to help them not harm them.^44^ Heart surgeons know this when, if a 4-year-old vigorously refuses the anaesthetic mask or cannula, they cancel non-urgent surgery.^45^ They refer the child for play therapy until the child can accept the frightening procedures in order to gain the benefits of better health.

Actual clinical practice also differs from legal age standards noted earlier when, in rare cases, doctors accept the refusal of a lifesaving heart transplant by children aged from about 6 years. This happens only after weeks of shared discussions and the child's name stays on the waiting list in case of a change of mind before it is too late. Clinicians must give scarce hearts to children who will most willingly cooperate with the essential, lifelong, daily follow-up care. These decisions respect young children's rational, informed decision making and capacity to consent or refuse in both a legal event and a personal process.

## Conclusion

Traditionally, adults have desired to protect children, from frightening details about serious risks of treatment, and the dangers of making the ‘wrong’ decision. They often believed young children lack informed bodily integrity and autonomy, the related knowledge to make serious decisions, and the courage to stand by them. During interviews, clinicians reported that many parents did not want their child to be informed before having heart surgery.^46^

Encouraged by newer insights and their professional experience, the clinicians persuaded parents to let them inform the children, having seen the long-term trauma endured by children who were not informed and prepared.^46^ They saw helping children to deal with worrying information as part of therapy.

Adults’ reservations about involving children in decisions relate to epistemic injustice, when disadvantaged groups are assumed to lack knowledge-related capacities. Their views are then ignored or disbelieved and dismissed. Their attempts to speak may seem impertinent.^47^ The erudite traditions of philosophy, law and medicine have an enduring history of epistemic superiority, which doctors are slowly changing.

The strongest deterrent to involving children in healthcare decisions may be financial, to prevent costly complaints and litigation, by ensuring that consent is legally valid and adult-centred. Yet even when others signify consent for them, it is still vital to listen to children and young people and inform and involve them in decision making as much as they are willing and able to be involved. This can be cost-effective in reducing risks of post traumatic stress disorder (PTSD) and episodes of time-consuming resistance by fearful children. The riskier and more complex the decision, the more important this involvement may be, to respect children's bodily integrity and autonomy and best interests.
